# Supplementary material for: The Role of Vegetation on the Dynamics of Water and Fire in the Cerrado Ecosystems: Implications for Management and Conservation
Source: Plants (Basel). 2020 Dec 18;9(12):1803. doi: 10.3390/plants9121803 (PMC7766540; doi:10.3390/plants9121803)
Supplement: Supplementary file 1 [file plants-09-01803-s001.pdf]

Review

Vegetation can play a fundamental role in protecting functioning of ecosystems and Cerrado conservation

Carlos A. Klink [1], Margarete N. Sato [1], Giovanna G. Cordeiro [1] and Maria Inês M. Ramos [2]

[1] Department of Ecology, University of Brasilia (UnB), Brasília, Brazil; carlosklink@gmail.com (C.K.); nsatoecologia@gmail.com (M.S.); giogomes.2402@gmail.com (G.C.)
[2] The World Bank; nesita03@gmail.com
* Correspondence: carlosklink@gmail.com

Supplementary Materials

Table S1. Brazil's policy instruments in ecosystem conservation, sustainable land use, and climate change mitigation for the Cerrado.

Policy instrument	Main objectives
Law for Native Vegetation Protection (The Forest Code)	The Forest Code establishes general regulations for protecting the vegetation in private lands, including the permanent protection areas (APP) and the Legal Reserves (RL). It also sets the general rules for wood supply and transportation, forest fires, and the establishment of economic incentives for forest conservation and restoration.
Rural Environmental Register (CAR)	The CAR is a nationwide electronic registry that is mandatory for all rural properties and possessions; CAR is key to the allocation of native vegetation in private lands for conservation and for monitoring the fulfilment of legal commitments. The purpose of this instrument is to integrate all the environmental information from these properties and rural possessions, composing a database for deforestation control, environmental monitoring, and economic planning. Registry in the CAR is also mandatory for land owners to gain access to the benefits provided under the Forest Code Law, such as (i) authorization for native vegetation suppression, (ii) participation in the Environmental Regularization Program (PRA), and (iii) access to agricultural credit lines.
Environmental Regularization Program (PRA)	The PRA is a specific instrument of the Forest Code to fulfil the compliance of those lands that were deforested on private properties before June 2008. It is the next step in the Forest Code implementation process for those rural properties that lack enough natural vegetation within their limits and have registered in CAR on time. PRA should allow a smoother path for environmental compliance of the property, including longer terms to promote natural vegetation restoration and the withholding of fines and administrative sanctions.

Environmental Reserve Quota (CRA)	The CRA is an instrument of the Forest Code with the purpose of facilitating the environmental regularization of rural properties by trading certificates of areas with preserved native vegetation. The compliance of the Legal Reserves can be achieved by the maintenance of native vegetation in other properties, reducing regularization costs, and promoting an incentive for properties with additional forest areas.
Action Plan for Prevention and Controlling of Deforestation and Fires (PPCerrado)	PPCerrado was created to reduce native vegetation deforestation and degradation and fostering ecosystem services. It also establishes that economic development should contemplates the conservation of biodiversity, water resources, and the cultural and natural heritage of traditional populations. The Plan builds on the successful implementation of the PPCDAm, which successfully allowed a 72% reduction of deforestation in the Amazon since 2004. The PPCerrado is structured on four axes of operation: territorial organization, monitoring and control, sustainable use, and economic instruments. PPCerrado also functions as a reference for the application of international resources and for the identification of priorities in national environmental policies.
Nationally Determined Contribution (NDC)	Brazil's NDC establishes and organizes the country's actions to reduce greenhouse gas emissions by 37% below 2005 levels in 2025. All policies, measures and actions to implement Brazil's NDC are carried out under the related legislation, instruments, and planning processes of the National Policy on Climate Change (Law 12.187/2009), the Forest Code (Law 12.651/2012), and the Law on the National System of Protected Areas (Law 9.985/2000). In December 2019, the Environmental Committee of the Brazilian Senate elaborated a thorough evaluation of the National Policy on Climate Change and its outcomes. Its main recommendations were to adjust the current law by introducing legislation text that reflects the Paris 2015 climate change agreement and the Brazilian NDC into the law, to include a long-term National Strategy to become zero-net emissions by 2050, to amplify civil society participation into policy making, to stipulate a timeframe and outcomes for deforestation control, among other recommendations. The bill with recommendations is to be appreciated by the Brazilian Senate.
National Plan for Restoration of Native Vegetation (Planaveg)	Planaveg is one of the instruments of the National Policy for the Restoration of the Native Vegetation (Proveg) that has the purpose of: (i) restoring the native vegetation of rural properties to meet compliance requirements of the Forest Code, and (ii) strengthening public policies, financial incentives, markets, restoration technologies, best agricultural and livestock practices, and other measures necessary for the restoration of native vegetation. The Proveg's target is to reach 12 million hectares restored throughout the country by 2030.
National System of Protected Areas (SNUC)	SNUC´s aims at (i) conserving the variety of biological species and genetic resources within the national territory; (ii) protecting endangered species; (iii) providing preservation and restoration for the diversity of natural ecosystems; (iv) promoting the use of nature

	conservation principles and practices within development processes; (v) providing means and incentives for scientific research and environmental monitoring; and (vi) protecting natural resources for the subsistence of traditional populations, respecting and valuing their knowledge and culture.
National Strategy for REDD+ (ENREDD+)	Developed by the United Nations Framework Convention on Climate Change (UNFCCC), REDD+ is an instrument designed to financially reward developing countries for their results related to: (i) deforestation emissions reduction; (ii) forest degradation emissions reduction; (iii) forest carbon stocks conservation; (iv) sustainable forests management; and (v) forest carbon stocks enhancement. The overall goal established by ENREDD + is to contribute to climate change mitigation, by coordinating and promoting synergies among the National Policy on Climate Change, the Forest Code, Action Plans in biomes and other relevant instruments, with a focus on prevention and control of deforestation, degradation forest recovery, and promotion of sustainable development.
Cerrado Monitoring Program (Prodes and DETER Cerrado)	Prodes Cerrado is a deforestation mapping project for the whole extension of the Cerrado. Its mains objectives are to (i) strengthen Brazil's institutional capacity to monitor deforestation, (ii) provide information on forest fire risks, and (iii) estimate the emission of GHG that comes from deforestation and fires in the Cerrado. The systematic monitoring of the Cerrado broadens the land cover data produced by the National Institute of Space Research (INPE). With the inclusion of the Cerrado in the monitoring programs, the mapping guarantees an information base about deforestation in natural vegetation areas for 73% of the Brazilian territory.
Low Carbon Agriculture Plan (*Plano ABC*)	The Low Carbon Agriculture Plan promotes actions to enhance the use of sustainable production technologies. ABC was built as part of Brazil's response to reduce the emission of greenhouse gases. Through credit programs nurtured by the Brazilian Development Bank (BNDES), the Plan integrates actions of the productive sector with federal, state, and municipal governments, rural communities, and civil society.
Ecological-economic zoning (ZEE)	ZEE is a planning instrument that establishes environmental measures and plans that secure quality biodiversity conservation and protection of soil and water resources. It aims to link and organize public and private agents in their decision making about programs, projects, and activities that benefit from natural resources. ZEE can be applied at the federal, state, or municipal levels, if social, economic, cultural, and environmental factors of each location are considered.

Table S2. Funds and financing opportunities for the Cerrado.

**Fund/financing source**	**Main objectives**
The Amazon Fund	The Amazon Fund was set to reward Brazilian success in reducing deforestation in the Amazon. Its main goal is to support, through non-reimbursable projects, the sustainable development in the region and the protection of native vegetation. Up to 20% of the resources can be applied to other Brazilian biomes and other tropical forests in the world.
The Climate Change Fund	The Climate Change Fund was built to support the deployment of Brazil's Climate Change Policy to achieve the country´s goals for reduction of greenhouse gases emissions. The resources for the Climate Change Fund come from (i) the oil exploration and production sector; (ii) the Annual Federal Budget; (iii) agreements, contracts, covenants, partnerships terms, and other similar instruments executed between public administration bodies and entities; (iv) donations by national and international entities, private or public; (v) loans from national and international financial institutions; (vi) reversal of annual provisions not applied; (vii) resources from financing interest and amortizations.
BNDES Ecological Restoration	Originally designed for the Atlantic Rainforest, this initiative aims at supporting non-reimbursable and reimbursable projects for restoration in all Brazilian biomes, except in the Amazon biome. The aim is to finance companies, farmers, and civil society projects to increase forest cover and strengthen the supply chain of the forest restoration sector.
Credit lines for restoration/BNDES	BNDES provides credit lines for small, medium, and larger farmers to achieve environmental regularization of their rural properties.
Environmental Fines Conversion Program (IBAMA)	The conversion of environmental fines was foreseen in Decree 6,514/2008. Decree 9,179/2017 altered the original decree and created the Environmental Fines Conversion Program, allowing the replacement of environmental fines by the provision of services for preservation, improvement, and recovery of the environment. Each federal environmental institution set its own procedures, following the main guidelines from Decree 9,179/2017.
Forest Invention Program (FIP-Cerrado)	The Forest Investment Program in Brazil is part of a wider portfolio of the Climate Investment Funds (CIF), which allocates $8 billion (USD) to accelerate climate action by empowering transformations in clean technology, energy access, climate resilience, and sustainable forests in developing and middle-income countries. The resources come from 14 donor countries, and it is managed by the World Bank. The funds are disbursed as grants, highly concessional loans, and risk mitigation instruments to recipient countries through multilateral development banks (MDBs).<br>The Brazilian program is focused on land use and land use change in the Cerrado biome.

Global Environment Facility (GEF-Matopiba)	The GEF-Matopiba promotes sustainable production and biodiversity conservation in the region known as Matopiba, which encompasses the states of Maranhão, Tocantins, Piauí and Bahia.
Green Climate Fund (GCF)	GCF is a financial mechanism established under the United Nations Framework Climate Change Convention (UNFCCC) to support developing countries in climate change mitigation and adaptation actions.
Plano Safra	The Plano Safra is the main allocation of the federal government to finance agriculture throughout the country. The aim is to support the agriculture and livestock production in Brazil.
The National Program for the Support of Family Agriculture (PRONAF)	PRONAF finances projects that generate income for family farmers and settlers of agrarian reform.
